# Bis[*N*-(pyridin-2-ylcarbon­yl)pyridine-2-carboximidato-κ^3^ *N*,*N*′,*N*′′]iron(III) tris­(cyanido-κ*C*)[*N*-(pyridin-2-ylcarbon­yl)pyridine-2-carboximidato-κ^3^ *N*,*N*′,*N*′′]ferrate(III) monohydrate

**DOI:** 10.1107/S1600536811046800

**Published:** 2011-11-09

**Authors:** Yunyun Xiao, Xiaoping Shen, Yizhi Li

**Affiliations:** a School of Chemistry and Chemical Engineering, Jiangsu University, Zhenjiang 212013, People’s Republic of China; b State Key Laboratory of Coordination Chemistry, Nanjing University, Nanjing 210093, People’s Republic of China

## Abstract

In the title compound, [Fe(C_12_H_8_N_3_O_2_)_2_][Fe(C_12_H_8_N_3_O_2_)(CN)_3_]·H_2_O, the Fe^3+^ ions in the cation and anion each lie in a slightly distorted octa­hedral coordination environment. The solvent water mol­ecule is disordered over three positions with occupancies of 0.401 (7), 0.322 (7) and 0.277 (6). The water content was confirmed by thermogravimetric data.

## Related literature

For the background to cyanide-bridged low-dimensional systems, see: Lescouëzec *et al.* (2005 ▶). For related structures, see: Lescouëzec *et al.* (2004 ▶); Wen *et al.* (2006 ▶); Wu (2009 ▶). 
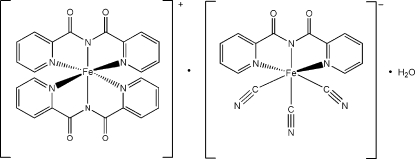


## Experimental

### 

#### Crystal data

[Fe(C_12_H_8_N_3_O_2_)_2_][Fe(C_12_H_8_N_3_O_2_)(CN)_3_]·H_2_O
*M* *_r_* = 886.42
Triclinic, 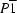

*a* = 9.6116 (12) Å
*b* = 14.2025 (13) Å
*c* = 15.1032 (16) Å
α = 98.154 (2)°
β = 99.645 (3)°
γ = 104.558 (2)°
*V* = 1930.4 (4) Å^3^
*Z* = 2
Mo *K*α radiation
μ = 0.82 mm^−1^
*T* = 291 K
0.28 × 0.24 × 0.22 mm

#### Data collection

Rigaku CCD area-detector diffractometer
Absorption correction: multi-scan (*ABSCOR*; Higashi, 1995 ▶) *T*_min_ = 0.803, *T*_max_ = 0.840
18046 measured reflections
6943 independent reflections
6052 reflections with *I* > 2σ(*I*)
*R*_int_ = 0.029

#### Refinement

*R*[*F* ^2^ > 2σ(*F* ^2^)] = 0.056
*wR*(*F* ^2^) = 0.132
*S* = 1.10
6943 reflections
562 parameters
1 restraint
H-atom parameters constrained
Δρ_max_ = 0.63 e Å^−3^
Δρ_min_ = −0.61 e Å^−3^

### 

Data collection: *CrystalClear* (Rigaku, 2008 ▶); cell refinement: *CrystalClear*; data reduction: *CrystalClear*; program(s) used to solve structure: *SHELXS97* (Sheldrick, 2008 ▶); program(s) used to refine structure: *SHELXL97* (Sheldrick, 2008 ▶); molecular graphics: *DIAMOND* (Brandenburg, 2006 ▶); software used to prepare material for publication: *SHELXTL* (Sheldrick, 2008 ▶).

## Supplementary Material

Crystal structure: contains datablock(s) I, global. DOI: 10.1107/S1600536811046800/yk2026sup1.cif

Structure factors: contains datablock(s) I. DOI: 10.1107/S1600536811046800/yk2026Isup2.hkl

Additional supplementary materials:  crystallographic information; 3D view; checkCIF report

## Figures and Tables

**Fig. 1.**
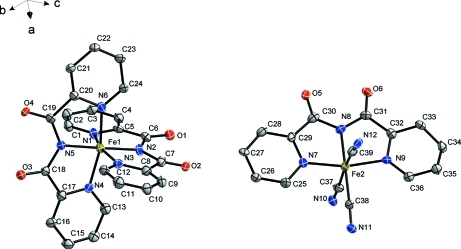
ORTEP diagram of an asymmetric unit of the title compound. Displacement ellipsoids are drawn at the 30% probability level. Hydrogen atoms and the disordered water molecule have been omitted for clarity.

## supplementary crystallographic information

### Comment

In recent years, have been developed new synthetic strategies to prepare cyano-bridged low-dimensional systems by using modified cyanometalates, [*M*(*L*)*_y_*(CN)*_x_*]^(^*^x^*^-m)-^ (*M* = first row transition metal ions and *L* = organic polydentate ligands), as multidentate ligands (Lescouëzec *et al.*, 2005), because these cyano-bridged bimetallic low-dimensional assemblies possess extraordinary magnetic properties such as SMM (single molecule magnets) and SCM (single chain magnets). For example, it was found that [Fe(bpca)(CN)_3_]^-^ {bpca = [*N*-(2-pyridylcarbonyl)pyridine-2-carboximidate} can coordinate to transition metal ions to form various polynuclear and one-dimensional structures with interesting magnetic behaviors (Lescouëzec *et al.*, 2004; Wen *et al.*, 2006). Recently, we had expected to obtain such low-dimensional systems using [Fe(bpca)(CN)_3_]^-^ and lanthanide metal ion like Tb^3+^ as the building blocks. However, an unexpected ion-paired compound of [Fe^III^(bpca)_2_][Fe^III^(bpca)(CN)_3_] instead of any [Fe(bpca)(CN)_3_]^-^/Tb^3+^ cyano-bridged assembly was obtained. Herein, the crystal structure of the prepared complex is presented.

The asymmetric unit of the title complex consists of a [Fe^III^(bpca)_2_]^+^ cation, a [Fe^III^(bpca)(CN)_3_]^-^ anion and one H_2_O molecule (Fig. 1). In [Fe^III^(bpca)_2_]^+^cation, the Fe^III^ ion is coordinated by six nitrogen atoms from two tridentate bpca ligands in a *mer*–mode, and exhibits a distorted octahedral coordination configuration. The Fe1—N bond lengths are in the range of 1.910 (3)–1.979 (3) Å, which is consistent with the values 1.900–1.977 Å reported for [Fe^III^(bpca)_2_]ClO_4_.CH_3_OH (Wu, 2009). In [Fe^III^(bpca)(CN)_3_]^-^ anion, the Fe^III^ ion is coordinated by three carbon atoms of cyanide groups and three N atoms from bpca ligand in a *mer*-arrangement, which results in a distorted octahedral environment around the Fe^III^ ion. The Fe2—N(bpca) bond distances vary in the range of 1.898 (3)–1.977 (3) Å, which are close to those (1.893 (2)–1.959 (2) Å) found in the complex of PPh_4_[Fe^III^(bpca)(CN)_3_].H_2_O (Lescouëzec *et al.*, 2004). The Fe2—*C*(cyano) bond lengths (1.922 (4)–2.000 (4) Å) are slightly longer than those (1.937 (3)–1.951 (3) Å) reported for PPh_4_[Fe^III^(bpca)(CN)_3_].H_2_O. The interstitial water molecule in the structure was found to be severely disordered and has been refined as disordered over three positions with occupancies of 0.401 (7), 0.322 (7) and 0.277 (6) for O7, O8 and O9, respectively.

### Experimental

A solution of Tb(NO_3_)_3_.6H_2_O (0.05 mmol) in water (10 ml) was added to a solution of Bu_4_N[Fe^III^(bpca)(CN)_3_] (0.05 mmol) in MeCN/H_2_O [4/1(V/V), 10 ml] mixture. The resulting solution was filtered and the filtrate was allowed to slow evaporation in dark at room temperature. Red block-shaped crystals suitable for single-crystal X-ray diffraction were obtained after two weeks. Anal. Calc. for C_39_H_26_Fe_2_N_12_O_7_: C, 52.85; H, 2.96; N, 18.96; Fe, 12.60%. Found: C, 53.04; H, 2.73; N, 19.11; Fe, 12.45%. The TGA curve shows 2.17% loss of mass, which is in good agreement with 2.03% calculated for one water molecule per asymmetric unit.

### Refinement

All non-H atoms were refined anisotropically. All H atoms including ligand and interstitial water were placed in calculated positions and with C—H = 0.93–0.97 Å, and with *U*_eq_ values set at 1.2–1.5 *U*_eq_ of the parent atoms. Each asymmetric unit contains one crystal water molecule, the water molecule is disordered over three positions with refined occupancies of 0.401 (7), 0.322 (7) and 0.277 (6).

### Crystal data

**Table:**

[Fe(C_12_H_8_N_3_O_2_)_2_][Fe(C_12_H_8_N_3_O_2_)(CN)_3_]·H_2_O	*Z* = 2
*M**_r_* = 886.42	*F*(000) = 904
Triclinic, *P*1	*D*_x_ = 1.525 Mg m^−^^3^
Hall symbol: -P 1	Mo *K*α radiation, λ = 0.71073 Å
*a* = 9.6116 (12) Å	Cell parameters from 3763 reflections
*b* = 14.2025 (13) Å	θ = 2.1–25.1°
*c* = 15.1032 (16) Å	µ = 0.82 mm^−^^1^
α = 98.154 (2)°	*T* = 291 K
β = 99.645 (3)°	Prism, red
γ = 104.558 (2)°	0.28 × 0.24 × 0.22 mm
*V* = 1930.4 (4) Å^3^	

### Data collection

**Table:**

Rigaku CCD area-detector diffractometer	6943 independent reflections
Radiation source: fine-focus sealed tube	6052 reflections with *I* > 2σ(*I*)
graphite	*R*_int_ = 0.029
φ and ω scans	θ_max_ = 25.3°, θ_min_ = 3.0°
Absorption correction: multi-scan (*ABSCOR*; Higashi, 1995)	*h* = −11→11
*T*_min_ = 0.803, *T*_max_ = 0.840	*k* = −14→17
18046 measured reflections	*l* = −18→17

### Refinement

**Table:**

Refinement on *F*^2^	Primary atom site location: structure-invariant direct methods
Least-squares matrix: full	Secondary atom site location: difference Fourier map
*R*[*F*^2^ > 2σ(*F*^2^)] = 0.056	Hydrogen site location: inferred from neighbouring sites
*wR*(*F*^2^) = 0.132	H-atom parameters constrained
*S* = 1.10	*w* = 1/[σ^2^(*F*_o_^2^) + (0.06*P*)^2^ + 1.99*P*] where *P* = (*F*_o_^2^ + 2*F*_c_^2^)/3
6943 reflections	(Δ/σ)_max_ < 0.001
562 parameters	Δρ_max_ = 0.63 e Å^−^^3^
1 restraint	Δρ_min_ = −0.61 e Å^−^^3^

### Special details

**Table:**

Geometry. All s.u.'s (except the s.u.'s in the dihedral angle between two l.s. planes) are estimated using the full covariance matrix. The cell s.u.'s are taken into account individually in the estimation of s.u.'s in distances, angles and torsion angles; correlations between s.u.'s in cell parameters are only used when they are defined by crystal symmetry. An approximate (isotropic) treatment of cell s.u.'s is used for estimating s.u.'s involving l.s. planes.
Refinement. Refinement of *F*^2^ against ALL reflections. The weighted *R*-factor *wR* and goodness of fit *S* are based on *F*^2^, conventional *R*-factors *R* are based on *F*, with *F* set to zero for negative *F*^2^. The threshold expression of *F*^2^ > σ(*F*^2^) is used only for calculating *R*-factors(gt) *etc*. and is not relevant to the choice of reflections for refinement. *R*-factors based on *F*^2^ are statistically about twice as large as those based on *F*, and *R*- factors based on ALL data will be even larger.

### Fractional atomic coordinates and isotropic or equivalent isotropic displacement parameters (Å^2^)

**Table:**

	*x*	*y*	*z*	*U*_iso_*/*U*_eq_	Occ. (<1)
C1	0.1550 (4)	0.5880 (3)	0.2213 (3)	0.0390 (8)	
H1	0.1786	0.6021	0.1665	0.047*	
C2	0.0448 (4)	0.6193 (3)	0.2510 (3)	0.0438 (9)	
H2	−0.0021	0.6576	0.2185	0.053*	
C3	0.0037 (4)	0.5941 (3)	0.3286 (3)	0.0450 (9)	
H3	−0.0770	0.6107	0.3456	0.054*	
C4	0.0787 (4)	0.5453 (3)	0.3816 (3)	0.0408 (8)	
H4	0.0568	0.5324	0.4372	0.049*	
C5	0.1900 (4)	0.5159 (3)	0.3475 (3)	0.0397 (8)	
C6	0.2753 (4)	0.4547 (3)	0.3944 (3)	0.0414 (8)	
C7	0.4915 (4)	0.3953 (2)	0.3852 (2)	0.0382 (8)	
C8	0.5828 (4)	0.3863 (3)	0.3157 (2)	0.0418 (8)	
C9	0.6924 (4)	0.3396 (3)	0.3248 (2)	0.0400 (8)	
H9	0.7134	0.3133	0.3768	0.048*	
C10	0.7703 (4)	0.3318 (3)	0.2579 (3)	0.0431 (9)	
H10	0.8507	0.3064	0.2676	0.052*	
C11	0.7332 (4)	0.3601 (3)	0.1778 (3)	0.0476 (9)	
H11	0.7813	0.3504	0.1302	0.057*	
C12	0.6182 (4)	0.4051 (3)	0.1698 (2)	0.0419 (8)	
H12	0.5937	0.4292	0.1169	0.050*	
C13	0.6159 (4)	0.6490 (3)	0.3617 (2)	0.0376 (8)	
H13	0.5935	0.6199	0.4108	0.045*	
C14	0.7276 (4)	0.7360 (3)	0.3758 (2)	0.0393 (8)	
H14	0.7752	0.7671	0.4355	0.047*	
C15	0.7696 (4)	0.7769 (3)	0.3067 (3)	0.0482 (9)	
H15	0.8500	0.8327	0.3173	0.058*	
C16	0.6909 (4)	0.7344 (3)	0.2202 (3)	0.0530 (10)	
H16	0.7148	0.7638	0.1715	0.064*	
C17	0.5797 (4)	0.6510 (3)	0.2040 (2)	0.0401 (8)	
C18	0.4896 (3)	0.5990 (2)	0.1128 (2)	0.0331 (7)	
C19	0.2682 (4)	0.4607 (3)	0.0464 (2)	0.0400 (8)	
C20	0.1788 (4)	0.3704 (3)	0.0731 (2)	0.0382 (8)	
C21	0.0660 (4)	0.3025 (3)	0.0180 (3)	0.0494 (10)	
H21	0.0379	0.3095	−0.0421	0.059*	
C22	−0.0153 (4)	0.2170 (3)	0.0490 (3)	0.0536 (11)	
H22	−0.0953	0.1690	0.0108	0.064*	
C23	0.0318 (4)	0.2118 (3)	0.1349 (2)	0.0433 (9)	
H23	−0.0168	0.1587	0.1582	0.052*	
C24	0.1537 (4)	0.2843 (3)	0.1922 (2)	0.0384 (8)	
H24	0.1832	0.2772	0.2522	0.046*	
C25	0.7555 (4)	0.1371 (3)	0.6227 (3)	0.0475 (9)	
H25	0.8535	0.1462	0.6497	0.057*	
C26	0.7204 (4)	0.1721 (3)	0.5429 (2)	0.0420 (8)	
H26	0.7948	0.2046	0.5165	0.050*	
C27	0.5740 (4)	0.1584 (3)	0.5024 (3)	0.0492 (10)	
H27	0.5505	0.1818	0.4490	0.059*	
C28	0.4628 (4)	0.1098 (3)	0.5417 (3)	0.0457 (9)	
H28	0.3648	0.1006	0.5147	0.055*	
C29	0.4979 (4)	0.0747 (2)	0.6215 (2)	0.0348 (7)	
C30	0.3941 (4)	0.0205 (3)	0.6703 (2)	0.0408 (8)	
C31	0.3943 (4)	−0.0594 (3)	0.8027 (3)	0.0455 (9)	
C32	0.5023 (4)	−0.0738 (3)	0.8766 (2)	0.0395 (8)	
C33	0.4645 (4)	−0.1290 (3)	0.9454 (3)	0.0423 (9)	
H33	0.3681	−0.1641	0.9439	0.051*	
C34	0.5839 (4)	−0.1271 (3)	1.0165 (3)	0.0394 (8)	
H34	0.5654	−0.1487	1.0699	0.047*	
C35	0.7131 (4)	−0.0965 (3)	1.0072 (3)	0.0404 (8)	
H35	0.7883	−0.1092	1.0469	0.048*	
C36	0.7472 (4)	−0.0450 (3)	0.9407 (2)	0.0400 (8)	
H36	0.8457	−0.0161	0.9414	0.048*	
C37	0.6896 (4)	0.1629 (3)	0.8446 (2)	0.0395 (8)	
C38	0.8851 (4)	0.0780 (3)	0.8024 (2)	0.0398 (8)	
C39	0.6958 (4)	−0.0899 (3)	0.7025 (3)	0.0420 (8)	
Fe1	0.38487 (6)	0.47999 (4)	0.23489 (3)	0.03707 (15)	
Fe2	0.67373 (5)	0.03413 (4)	0.77245 (4)	0.03803 (15)	
N1	0.2297 (3)	0.5375 (2)	0.2693 (2)	0.0386 (7)	
N2	0.3856 (3)	0.4435 (2)	0.3520 (2)	0.0390 (7)	
N3	0.5435 (3)	0.4141 (2)	0.23525 (19)	0.0354 (6)	
N4	0.5388 (3)	0.6062 (2)	0.27491 (19)	0.0363 (6)	
N5	0.3787 (3)	0.5174 (2)	0.11802 (19)	0.0368 (6)	
N6	0.2278 (3)	0.3621 (2)	0.16414 (19)	0.0358 (6)	
N7	0.6443 (3)	0.0884 (2)	0.6620 (2)	0.0388 (7)	
N8	0.4650 (3)	−0.0055 (2)	0.7452 (2)	0.0421 (7)	
N9	0.6479 (3)	−0.0345 (2)	0.8762 (2)	0.0425 (7)	
N10	0.7025 (3)	0.2397 (2)	0.8813 (2)	0.0392 (7)	
N11	1.0150 (3)	0.1090 (2)	0.8213 (2)	0.0432 (7)	
N12	0.7123 (3)	−0.1607 (2)	0.6665 (2)	0.0424 (7)	
O1	0.2532 (3)	0.43098 (18)	0.46654 (17)	0.0423 (6)	
O2	0.5156 (3)	0.37206 (17)	0.45674 (16)	0.0384 (6)	
O3	0.5171 (3)	0.62797 (19)	0.04174 (17)	0.0434 (6)	
O4	0.2354 (3)	0.47773 (19)	−0.03012 (17)	0.0484 (7)	
O5	0.2636 (3)	0.00215 (18)	0.64104 (17)	0.0413 (6)	
O6	0.2625 (3)	−0.09010 (18)	0.79723 (17)	0.0419 (6)	
O7	1.0200 (6)	0.6983 (5)	0.5415 (4)	0.0416 (19)	0.401 (7)
H7D	0.9470	0.6556	0.5664	0.050*	0.401 (7)
H7A	0.9659	0.7370	0.5352	0.050*	0.401 (7)
O8	0.0047 (9)	0.8413 (6)	0.6309 (6)	0.058 (3)	0.322 (7)
H8A	−0.0198	0.8312	0.6810	0.069*	0.322 (7)
H8B	0.0875	0.8849	0.6421	0.069*	0.322 (7)
O9	−0.0225 (9)	0.0250 (6)	0.5151 (6)	0.042 (3)	0.277 (6)
H9A	0.0440	0.0053	0.5451	0.050*	0.277 (6)
H9B	0.0062	0.0784	0.4956	0.050*	0.277 (6)

### Atomic displacement parameters (Å^2^)

**Table:**

	*U*^11^	*U*^22^	*U*^33^	*U*^12^	*U*^13^	*U*^23^
C1	0.043 (2)	0.0382 (19)	0.044 (2)	0.0177 (16)	0.0144 (16)	0.0162 (16)
C2	0.045 (2)	0.045 (2)	0.048 (2)	0.0183 (17)	0.0137 (17)	0.0134 (17)
C3	0.0360 (19)	0.051 (2)	0.047 (2)	0.0140 (17)	0.0061 (16)	0.0066 (18)
C4	0.045 (2)	0.0350 (18)	0.043 (2)	0.0060 (16)	0.0128 (16)	0.0119 (15)
C5	0.0242 (16)	0.0396 (19)	0.051 (2)	−0.0031 (14)	0.0082 (15)	0.0149 (16)
C6	0.039 (2)	0.0323 (18)	0.048 (2)	−0.0011 (15)	0.0074 (17)	0.0155 (16)
C7	0.046 (2)	0.0321 (17)	0.0335 (19)	0.0112 (15)	0.0003 (15)	0.0048 (14)
C8	0.042 (2)	0.042 (2)	0.0373 (19)	0.0109 (16)	0.0001 (16)	0.0048 (15)
C9	0.050 (2)	0.0384 (19)	0.0352 (19)	0.0128 (16)	0.0113 (16)	0.0138 (15)
C10	0.047 (2)	0.0387 (19)	0.045 (2)	0.0148 (17)	0.0105 (17)	0.0098 (16)
C11	0.052 (2)	0.050 (2)	0.041 (2)	0.0107 (18)	0.0165 (18)	0.0052 (17)
C12	0.0341 (18)	0.049 (2)	0.0373 (19)	0.0028 (16)	0.0054 (15)	0.0115 (16)
C13	0.047 (2)	0.0344 (18)	0.0318 (18)	0.0128 (16)	0.0126 (15)	0.0021 (14)
C14	0.049 (2)	0.0341 (18)	0.0343 (18)	0.0173 (16)	0.0054 (16)	0.0000 (14)
C15	0.044 (2)	0.046 (2)	0.043 (2)	−0.0012 (17)	0.0067 (17)	−0.0007 (17)
C16	0.045 (2)	0.057 (2)	0.041 (2)	−0.0077 (19)	−0.0025 (17)	0.0113 (18)
C17	0.044 (2)	0.0381 (19)	0.0361 (19)	0.0073 (16)	0.0102 (16)	0.0056 (15)
C18	0.0288 (16)	0.0315 (17)	0.0397 (19)	0.0094 (14)	0.0051 (14)	0.0108 (14)
C19	0.0319 (18)	0.046 (2)	0.0382 (19)	0.0000 (15)	0.0065 (15)	0.0156 (16)
C20	0.0391 (19)	0.0354 (18)	0.0351 (18)	0.0006 (15)	0.0071 (15)	0.0099 (15)
C21	0.0355 (19)	0.047 (2)	0.054 (2)	−0.0033 (16)	−0.0108 (17)	0.0239 (18)
C22	0.049 (2)	0.043 (2)	0.047 (2)	−0.0135 (18)	−0.0129 (18)	0.0144 (18)
C23	0.0279 (17)	0.053 (2)	0.041 (2)	−0.0017 (16)	0.0036 (15)	0.0118 (17)
C24	0.0300 (17)	0.046 (2)	0.0337 (18)	0.0002 (15)	0.0013 (14)	0.0153 (15)
C25	0.0364 (19)	0.054 (2)	0.046 (2)	0.0009 (17)	0.0013 (16)	0.0196 (18)
C26	0.0396 (19)	0.0387 (19)	0.0385 (19)	−0.0039 (15)	0.0027 (16)	0.0117 (16)
C27	0.048 (2)	0.0340 (19)	0.044 (2)	−0.0158 (17)	−0.0093 (17)	0.0110 (16)
C28	0.0366 (19)	0.051 (2)	0.043 (2)	0.0137 (17)	−0.0078 (16)	0.0066 (17)
C29	0.0395 (18)	0.0233 (15)	0.0383 (18)	0.0101 (14)	−0.0045 (15)	0.0077 (13)
C30	0.036 (2)	0.044 (2)	0.0392 (19)	0.0151 (16)	−0.0025 (16)	0.0021 (16)
C31	0.0298 (19)	0.048 (2)	0.049 (2)	−0.0023 (16)	−0.0001 (16)	0.0144 (18)
C32	0.0388 (19)	0.0353 (18)	0.043 (2)	0.0045 (15)	0.0073 (16)	0.0153 (15)
C33	0.0361 (19)	0.0361 (18)	0.046 (2)	−0.0071 (15)	0.0068 (16)	0.0138 (16)
C34	0.043 (2)	0.0379 (18)	0.047 (2)	0.0179 (16)	0.0142 (16)	0.0225 (16)
C35	0.0364 (19)	0.044 (2)	0.046 (2)	0.0155 (16)	0.0126 (16)	0.0154 (17)
C36	0.0347 (18)	0.0400 (19)	0.040 (2)	0.0050 (15)	0.0004 (15)	0.0102 (16)
C37	0.0376 (19)	0.038 (2)	0.0346 (18)	−0.0028 (15)	0.0112 (15)	0.0016 (16)
C38	0.0336 (19)	0.048 (2)	0.0309 (18)	−0.0017 (16)	0.0033 (14)	0.0135 (15)
C39	0.042 (2)	0.041 (2)	0.041 (2)	0.0155 (17)	−0.0052 (16)	0.0090 (17)
Fe1	0.0397 (3)	0.0324 (3)	0.0388 (3)	0.0091 (2)	0.0052 (2)	0.0116 (2)
Fe2	0.0321 (3)	0.0352 (3)	0.0431 (3)	0.0053 (2)	−0.0012 (2)	0.0143 (2)
N1	0.0465 (17)	0.0322 (15)	0.0396 (16)	0.0128 (13)	0.0112 (13)	0.0097 (13)
N2	0.0555 (19)	0.0277 (14)	0.0374 (16)	0.0146 (13)	0.0101 (14)	0.0122 (12)
N3	0.0400 (16)	0.0307 (14)	0.0310 (15)	0.0077 (12)	0.0023 (12)	0.0013 (11)
N4	0.0395 (16)	0.0348 (15)	0.0333 (15)	0.0111 (12)	0.0018 (12)	0.0077 (12)
N5	0.0340 (15)	0.0416 (16)	0.0317 (15)	0.0039 (13)	0.0054 (12)	0.0112 (12)
N6	0.0437 (16)	0.0303 (14)	0.0325 (15)	0.0055 (12)	0.0109 (13)	0.0086 (12)
N7	0.0421 (17)	0.0344 (15)	0.0377 (16)	0.0062 (13)	0.0032 (13)	0.0140 (12)
N8	0.0297 (15)	0.0483 (18)	0.0431 (17)	0.0058 (13)	−0.0033 (13)	0.0137 (14)
N9	0.0295 (15)	0.0454 (17)	0.0415 (17)	−0.0056 (13)	−0.0046 (13)	0.0155 (14)
N10	0.0435 (17)	0.0392 (17)	0.0380 (16)	0.0100 (13)	0.0139 (13)	0.0144 (14)
N11	0.0376 (18)	0.0438 (17)	0.0417 (17)	−0.0027 (14)	0.0083 (14)	0.0128 (14)
N12	0.0400 (17)	0.0442 (18)	0.0394 (17)	0.0157 (14)	−0.0087 (13)	0.0093 (14)
O1	0.0443 (14)	0.0417 (14)	0.0440 (15)	0.0134 (11)	0.0117 (12)	0.0133 (12)
O2	0.0415 (13)	0.0382 (13)	0.0331 (13)	0.0117 (11)	−0.0033 (10)	0.0115 (10)
O3	0.0399 (14)	0.0462 (14)	0.0389 (14)	0.0025 (11)	0.0037 (11)	0.0146 (11)
O4	0.0452 (15)	0.0468 (15)	0.0393 (14)	0.0009 (12)	−0.0175 (11)	0.0166 (12)
O5	0.0393 (14)	0.0379 (13)	0.0449 (14)	0.0123 (11)	−0.0044 (11)	0.0150 (11)
O6	0.0384 (14)	0.0379 (13)	0.0455 (14)	−0.0001 (11)	0.0125 (11)	0.0105 (11)
O7	0.021 (3)	0.050 (4)	0.044 (4)	0.002 (3)	−0.005 (2)	0.009 (3)
O8	0.041 (5)	0.054 (5)	0.066 (6)	−0.013 (4)	0.013 (4)	0.016 (4)
O9	0.031 (5)	0.040 (6)	0.042 (6)	0.003 (4)	−0.010 (4)	0.003 (4)

### Geometric parameters (Å, °)

**Table:**

C1—N1	1.338 (4)	C24—N6	1.321 (4)
C1—C2	1.363 (5)	C24—H24	0.9300
C1—H1	0.9300	C25—C26	1.390 (5)
C2—C3	1.364 (5)	C25—N7	1.390 (5)
C2—H2	0.9300	C25—H25	0.9300
C3—C4	1.364 (5)	C26—C27	1.390 (5)
C3—H3	0.9300	C26—H26	0.9300
C4—C5	1.390 (5)	C27—C28	1.390 (6)
C4—H4	0.9300	C27—H27	0.9300
C5—N1	1.353 (5)	C28—C29	1.390 (5)
C5—C6	1.506 (5)	C28—H28	0.9300
C6—O1	1.221 (4)	C29—N7	1.390 (4)
C6—N2	1.357 (5)	C29—C30	1.467 (5)
C7—O2	1.177 (4)	C30—O5	1.206 (4)
C7—N2	1.426 (5)	C30—N8	1.366 (4)
C7—C8	1.489 (5)	C31—O6	1.216 (4)
C8—N3	1.354 (5)	C31—N8	1.387 (5)
C8—C9	1.378 (5)	C31—C32	1.465 (5)
C9—C10	1.363 (5)	C32—N9	1.372 (5)
C9—H9	0.9300	C32—C33	1.429 (5)
C10—C11	1.348 (5)	C33—C34	1.426 (5)
C10—H10	0.9300	C33—H33	0.9300
C11—C12	1.406 (5)	C34—C35	1.247 (5)
C11—H11	0.9300	C34—H34	0.9300
C12—N3	1.326 (5)	C35—C36	1.361 (5)
C12—H12	0.9300	C35—H35	0.9300
C13—N4	1.363 (4)	C36—N9	1.297 (4)
C13—C14	1.380 (5)	C36—H36	0.9300
C13—H13	0.9300	C37—N10	1.117 (4)
C14—C15	1.336 (5)	C37—Fe2	1.948 (4)
C14—H14	0.9300	C38—N11	1.185 (4)
C15—C16	1.364 (5)	C38—Fe2	1.922 (4)
C15—H15	0.9300	C39—N12	1.134 (5)
C16—C17	1.341 (5)	C39—Fe2	2.000 (4)
C16—H16	0.9300	Fe1—N5	1.910 (3)
C17—N4	1.384 (4)	Fe1—N2	1.911 (3)
C17—C18	1.478 (5)	Fe1—N4	1.956 (3)
C18—O3	1.247 (4)	Fe1—N6	1.976 (3)
C18—N5	1.385 (4)	Fe1—N1	1.977 (3)
C19—O4	1.221 (4)	Fe1—N3	1.979 (3)
C19—N5	1.373 (4)	Fe2—N8	1.898 (3)
C19—C20	1.500 (5)	Fe2—N7	1.942 (3)
C20—C21	1.320 (5)	Fe2—N9	1.977 (3)
C20—N6	1.409 (4)	O7—H7D	0.9700
C21—C22	1.453 (5)	O7—H7A	0.8500
C21—H21	0.9300	O8—H8A	0.8500
C22—C23	1.319 (5)	O8—H8B	0.8500
C22—H22	0.9300	O9—O9^i^	1.014 (16)
C23—C24	1.409 (5)	O9—H9A	0.8500
C23—H23	0.9300	O9—H9B	0.8501
			
N1—C1—C2	121.5 (3)	O5—C30—N8	128.4 (4)
N1—C1—H1	119.3	O5—C30—C29	119.7 (3)
C2—C1—H1	119.3	N8—C30—C29	111.8 (3)
C1—C2—C3	119.7 (4)	O6—C31—N8	127.4 (3)
C1—C2—H2	120.2	O6—C31—C32	122.3 (3)
C3—C2—H2	120.2	N8—C31—C32	110.3 (3)
C2—C3—C4	121.3 (4)	N9—C32—C33	119.0 (3)
C2—C3—H3	119.4	N9—C32—C31	116.9 (3)
C4—C3—H3	119.4	C33—C32—C31	124.0 (3)
C3—C4—C5	115.8 (3)	C34—C33—C32	115.8 (3)
C3—C4—H4	122.1	C34—C33—H33	122.1
C5—C4—H4	122.1	C32—C33—H33	122.1
N1—C5—C4	123.8 (3)	C35—C34—C33	120.0 (3)
N1—C5—C6	114.0 (3)	C35—C34—H34	120.0
C4—C5—C6	122.2 (3)	C33—C34—H34	120.0
O1—C6—N2	127.6 (3)	C34—C35—C36	121.9 (4)
O1—C6—C5	121.4 (3)	C34—C35—H35	119.1
N2—C6—C5	110.5 (3)	C36—C35—H35	119.1
O2—C7—N2	128.9 (3)	N9—C36—C35	122.7 (3)
O2—C7—C8	123.1 (3)	N9—C36—H36	118.7
N2—C7—C8	107.8 (3)	C35—C36—H36	118.7
N3—C8—C9	118.6 (3)	N10—C37—Fe2	175.1 (3)
N3—C8—C7	117.9 (3)	N11—C38—Fe2	177.2 (3)
C9—C8—C7	122.9 (3)	N12—C39—Fe2	176.8 (3)
C10—C9—C8	120.3 (3)	N5—Fe1—N2	178.25 (13)
C10—C9—H9	119.9	N5—Fe1—N4	82.70 (12)
C8—C9—H9	119.9	N2—Fe1—N4	97.75 (12)
C11—C10—C9	121.3 (4)	N5—Fe1—N6	82.91 (12)
C11—C10—H10	119.3	N2—Fe1—N6	96.64 (12)
C9—C10—H10	119.3	N4—Fe1—N6	165.61 (12)
C10—C11—C12	116.7 (4)	N5—Fe1—N1	96.89 (12)
C10—C11—H11	121.6	N2—Fe1—N1	81.40 (12)
C12—C11—H11	121.6	N4—Fe1—N1	93.56 (12)
N3—C12—C11	122.0 (3)	N6—Fe1—N1	88.18 (12)
N3—C12—H12	119.0	N5—Fe1—N3	99.12 (12)
C11—C12—H12	119.0	N2—Fe1—N3	82.59 (12)
N4—C13—C14	119.3 (3)	N4—Fe1—N3	87.56 (12)
N4—C13—H13	120.3	N6—Fe1—N3	94.70 (12)
C14—C13—H13	120.3	N1—Fe1—N3	163.96 (12)
C15—C14—C13	122.3 (3)	N8—Fe2—C38	178.23 (16)
C15—C14—H14	118.8	N8—Fe2—N7	82.21 (13)
C13—C14—H14	118.8	C38—Fe2—N7	97.87 (14)
C14—C15—C16	117.9 (4)	N8—Fe2—C37	94.17 (15)
C14—C15—H15	121.1	C38—Fe2—C37	84.06 (16)
C16—C15—H15	121.1	N7—Fe2—C37	89.14 (14)
C17—C16—C15	121.4 (4)	N8—Fe2—N9	83.05 (12)
C17—C16—H16	119.3	C38—Fe2—N9	96.90 (13)
C15—C16—H16	119.3	N7—Fe2—N9	165.21 (12)
C16—C17—N4	121.0 (3)	C37—Fe2—N9	93.16 (14)
C16—C17—C18	125.2 (3)	N8—Fe2—C39	95.83 (15)
N4—C17—C18	113.8 (3)	C38—Fe2—C39	85.94 (16)
O3—C18—N5	126.7 (3)	N7—Fe2—C39	91.68 (14)
O3—C18—C17	121.3 (3)	C37—Fe2—C39	169.99 (15)
N5—C18—C17	112.0 (3)	N9—Fe2—C39	88.59 (15)
O4—C19—N5	128.0 (3)	C1—N1—C5	117.8 (3)
O4—C19—C20	120.3 (3)	C1—N1—Fe1	127.5 (2)
N5—C19—C20	111.7 (3)	C5—N1—Fe1	114.5 (2)
C21—C20—N6	121.3 (3)	C6—N2—C7	123.1 (3)
C21—C20—C19	124.9 (3)	C6—N2—Fe1	118.2 (2)
N6—C20—C19	113.7 (3)	C7—N2—Fe1	118.2 (2)
C20—C21—C22	121.7 (3)	C12—N3—C8	120.6 (3)
C20—C21—H21	119.2	C12—N3—Fe1	126.2 (2)
C22—C21—H21	119.2	C8—N3—Fe1	112.9 (2)
C23—C22—C21	115.6 (3)	C13—N4—C17	117.9 (3)
C23—C22—H22	122.2	C13—N4—Fe1	127.7 (2)
C21—C22—H22	122.2	C17—N4—Fe1	114.1 (2)
C22—C23—C24	121.9 (3)	C19—N5—C18	125.6 (3)
C22—C23—H23	119.1	C19—N5—Fe1	117.9 (2)
C24—C23—H23	119.1	C18—N5—Fe1	116.5 (2)
N6—C24—C23	122.5 (3)	C24—N6—C20	116.9 (3)
N6—C24—H24	118.7	C24—N6—Fe1	129.6 (2)
C23—C24—H24	118.7	C20—N6—Fe1	113.0 (2)
C26—C25—N7	120.0 (3)	C25—N7—C29	120.0 (3)
C26—C25—H25	120.0	C25—N7—Fe2	125.4 (2)
N7—C25—H25	120.0	C29—N7—Fe2	114.6 (2)
C25—C26—C27	120.0 (3)	C30—N8—C31	124.3 (3)
C25—C26—H26	120.0	C30—N8—Fe2	118.0 (3)
C27—C26—H26	120.0	C31—N8—Fe2	117.7 (2)
C28—C27—C26	120.0 (3)	C36—N9—C32	119.0 (3)
C28—C27—H27	120.0	C36—N9—Fe2	129.0 (3)
C26—C27—H27	120.0	C32—N9—Fe2	112.0 (2)
C27—C28—C29	120.0 (3)	H7D—O7—H7A	90.2
C27—C28—H28	120.0	H8A—O8—H8B	109.5
C29—C28—H28	120.0	O9^i^—O9—H9A	57.9
C28—C29—N7	120.0 (3)	O9^i^—O9—H9B	107.3
C28—C29—C30	126.7 (3)	H9A—O9—H9B	116.4
N7—C29—C30	113.3 (3)		
			
N1—C1—C2—C3	−3.7 (6)	N4—Fe1—N3—C8	92.3 (2)
C1—C2—C3—C4	5.6 (6)	N6—Fe1—N3—C8	−102.0 (2)
C2—C3—C4—C5	−5.2 (6)	N1—Fe1—N3—C8	−2.1 (6)
C3—C4—C5—N1	3.3 (5)	C14—C13—N4—C17	2.0 (5)
C3—C4—C5—C6	−175.4 (3)	C14—C13—N4—Fe1	175.9 (2)
N1—C5—C6—O1	178.8 (3)	C16—C17—N4—C13	−0.7 (5)
C4—C5—C6—O1	−2.5 (5)	C18—C17—N4—C13	180.0 (3)
N1—C5—C6—N2	6.6 (4)	C16—C17—N4—Fe1	−175.3 (3)
C4—C5—C6—N2	−174.7 (3)	C18—C17—N4—Fe1	5.3 (4)
O2—C7—C8—N3	179.1 (3)	N5—Fe1—N4—C13	178.0 (3)
N2—C7—C8—N3	−6.1 (4)	N2—Fe1—N4—C13	−0.3 (3)
O2—C7—C8—C9	7.7 (6)	N6—Fe1—N4—C13	178.0 (4)
N2—C7—C8—C9	−177.4 (3)	N1—Fe1—N4—C13	81.5 (3)
N3—C8—C9—C10	7.3 (5)	N3—Fe1—N4—C13	−82.5 (3)
C7—C8—C9—C10	178.6 (3)	N5—Fe1—N4—C17	−7.9 (3)
C8—C9—C10—C11	−6.7 (6)	N2—Fe1—N4—C17	173.8 (2)
C9—C10—C11—C12	4.9 (6)	N6—Fe1—N4—C17	−7.9 (6)
C10—C11—C12—N3	−3.9 (6)	N1—Fe1—N4—C17	−104.4 (3)
N4—C13—C14—C15	−4.4 (6)	N3—Fe1—N4—C17	91.6 (3)
C13—C14—C15—C16	5.0 (6)	O4—C19—N5—C18	−10.3 (6)
C14—C15—C16—C17	−3.6 (7)	C20—C19—N5—C18	172.4 (3)
C15—C16—C17—N4	1.4 (7)	O4—C19—N5—Fe1	171.3 (3)
C15—C16—C17—C18	−179.3 (4)	C20—C19—N5—Fe1	−5.9 (4)
C16—C17—C18—O3	3.8 (6)	O3—C18—N5—C19	−8.3 (6)
N4—C17—C18—O3	−176.9 (3)	C17—C18—N5—C19	172.8 (3)
C16—C17—C18—N5	−177.3 (4)	O3—C18—N5—Fe1	170.1 (3)
N4—C17—C18—N5	2.1 (4)	C17—C18—N5—Fe1	−8.8 (4)
O4—C19—C20—C21	2.8 (6)	N4—Fe1—N5—C19	−172.1 (3)
N5—C19—C20—C21	−179.7 (4)	N6—Fe1—N5—C19	7.9 (3)
O4—C19—C20—N6	−178.5 (3)	N1—Fe1—N5—C19	−79.4 (3)
N5—C19—C20—N6	−1.0 (5)	N3—Fe1—N5—C19	101.6 (3)
N6—C20—C21—C22	1.4 (6)	N4—Fe1—N5—C18	9.4 (2)
C19—C20—C21—C22	−180.0 (4)	N6—Fe1—N5—C18	−170.6 (3)
C20—C21—C22—C23	−0.5 (7)	N1—Fe1—N5—C18	102.1 (3)
C21—C22—C23—C24	−0.4 (6)	N3—Fe1—N5—C18	−76.9 (3)
C22—C23—C24—N6	0.4 (6)	C23—C24—N6—C20	0.5 (5)
N7—C25—C26—C27	0.0 (6)	C23—C24—N6—Fe1	171.9 (3)
C25—C26—C27—C28	0.0 (6)	C21—C20—N6—C24	−1.4 (5)
C26—C27—C28—C29	0.0 (6)	C19—C20—N6—C24	179.9 (3)
C27—C28—C29—N7	0.0 (5)	C21—C20—N6—Fe1	−174.2 (3)
C27—C28—C29—C30	−178.7 (4)	C19—C20—N6—Fe1	7.0 (4)
C28—C29—C30—O5	0.2 (6)	N5—Fe1—N6—C24	−179.8 (3)
N7—C29—C30—O5	−178.6 (3)	N2—Fe1—N6—C24	−1.5 (3)
C28—C29—C30—N8	178.4 (3)	N4—Fe1—N6—C24	−179.8 (4)
N7—C29—C30—N8	−0.4 (4)	N1—Fe1—N6—C24	−82.6 (3)
O6—C31—C32—N9	−179.1 (4)	N3—Fe1—N6—C24	81.6 (3)
N8—C31—C32—N9	−0.6 (5)	N5—Fe1—N6—C20	−8.0 (2)
O6—C31—C32—C33	3.6 (6)	N2—Fe1—N6—C20	170.3 (2)
N8—C31—C32—C33	−177.9 (4)	N4—Fe1—N6—C20	−8.1 (6)
N9—C32—C33—C34	8.4 (5)	N1—Fe1—N6—C20	89.1 (2)
C31—C32—C33—C34	−174.3 (4)	N3—Fe1—N6—C20	−106.7 (2)
C32—C33—C34—C35	−14.2 (5)	C26—C25—N7—C29	0.0 (6)
C33—C34—C35—C36	14.4 (6)	C26—C25—N7—Fe2	179.5 (3)
C34—C35—C36—N9	−8.5 (6)	C28—C29—N7—C25	0.0 (5)
C2—C1—N1—C5	1.7 (5)	C30—C29—N7—C25	178.8 (3)
C2—C1—N1—Fe1	176.5 (3)	C28—C29—N7—Fe2	−179.6 (3)
C4—C5—N1—C1	−1.6 (5)	C30—C29—N7—Fe2	−0.7 (4)
C6—C5—N1—C1	177.2 (3)	N8—Fe2—N7—C25	−178.4 (3)
C4—C5—N1—Fe1	−177.0 (3)	C38—Fe2—N7—C25	3.4 (3)
C6—C5—N1—Fe1	1.7 (4)	C37—Fe2—N7—C25	87.3 (3)
N5—Fe1—N1—C1	−1.8 (3)	N9—Fe2—N7—C25	−173.6 (5)
N2—Fe1—N1—C1	178.5 (3)	C39—Fe2—N7—C25	−82.7 (3)
N4—Fe1—N1—C1	81.2 (3)	N8—Fe2—N7—C29	1.1 (2)
N6—Fe1—N1—C1	−84.5 (3)	C38—Fe2—N7—C29	−177.1 (3)
N3—Fe1—N1—C1	174.8 (4)	C37—Fe2—N7—C29	−93.2 (3)
N5—Fe1—N1—C5	173.1 (2)	N9—Fe2—N7—C29	6.0 (7)
N2—Fe1—N1—C5	−6.5 (2)	C39—Fe2—N7—C29	96.8 (3)
N4—Fe1—N1—C5	−103.8 (2)	O5—C30—N8—C31	−0.4 (6)
N6—Fe1—N1—C5	90.5 (2)	C29—C30—N8—C31	−178.4 (3)
N3—Fe1—N1—C5	−10.3 (6)	O5—C30—N8—Fe2	179.4 (3)
O1—C6—N2—C7	3.6 (6)	C29—C30—N8—Fe2	1.4 (4)
C5—C6—N2—C7	175.2 (3)	O6—C31—N8—C30	−1.2 (7)
O1—C6—N2—Fe1	176.0 (3)	C32—C31—N8—C30	−179.6 (3)
C5—C6—N2—Fe1	−12.5 (4)	O6—C31—N8—Fe2	179.0 (3)
O2—C7—N2—C6	−12.3 (6)	C32—C31—N8—Fe2	0.7 (4)
C8—C7—N2—C6	173.2 (3)	N7—Fe2—N8—C30	−1.4 (3)
O2—C7—N2—Fe1	175.4 (3)	C37—Fe2—N8—C30	87.1 (3)
C8—C7—N2—Fe1	0.9 (4)	N9—Fe2—N8—C30	179.8 (3)
N4—Fe1—N2—C6	103.4 (3)	C39—Fe2—N8—C30	−92.3 (3)
N6—Fe1—N2—C6	−76.2 (3)	N7—Fe2—N8—C31	178.4 (3)
N1—Fe1—N2—C6	10.9 (3)	C37—Fe2—N8—C31	−93.1 (3)
N3—Fe1—N2—C6	−170.1 (3)	N9—Fe2—N8—C31	−0.4 (3)
N4—Fe1—N2—C7	−83.9 (3)	C39—Fe2—N8—C31	87.4 (3)
N6—Fe1—N2—C7	96.5 (3)	C35—C36—N9—C32	2.4 (6)
N1—Fe1—N2—C7	−176.4 (3)	C35—C36—N9—Fe2	−178.5 (3)
N3—Fe1—N2—C7	2.6 (2)	C33—C32—N9—C36	−3.0 (5)
C11—C12—N3—C8	4.8 (5)	C31—C32—N9—C36	179.6 (3)
C11—C12—N3—Fe1	177.5 (3)	C33—C32—N9—Fe2	177.8 (3)
C9—C8—N3—C12	−6.3 (5)	C31—C32—N9—Fe2	0.3 (4)
C7—C8—N3—C12	−178.1 (3)	N8—Fe2—N9—C36	−179.1 (4)
C9—C8—N3—Fe1	−180.0 (3)	C38—Fe2—N9—C36	−0.9 (4)
C7—C8—N3—Fe1	8.3 (4)	N7—Fe2—N9—C36	176.1 (4)
N5—Fe1—N3—C12	1.2 (3)	C37—Fe2—N9—C36	−85.3 (4)
N2—Fe1—N3—C12	−179.1 (3)	C39—Fe2—N9—C36	84.9 (4)
N4—Fe1—N3—C12	−81.0 (3)	N8—Fe2—N9—C32	0.0 (3)
N6—Fe1—N3—C12	84.8 (3)	C38—Fe2—N9—C32	178.3 (3)
N1—Fe1—N3—C12	−175.4 (4)	N7—Fe2—N9—C32	−4.8 (7)
N5—Fe1—N3—C8	174.5 (2)	C37—Fe2—N9—C32	93.9 (3)
N2—Fe1—N3—C8	−5.9 (2)	C39—Fe2—N9—C32	−96.0 (3)

Symmetry codes: (i) −*x*, −*y*, −*z*+1.
